# Mutational landscape and clinical outcome of pediatric acute myeloid leukemia with 11q23/ *KMT2A* rearrangements

**DOI:** 10.1002/cam4.5026

**Published:** 2022-07-14

**Authors:** Ka‐Yuk Yuen, Yong Liu, Yong‐Zhuo Zhou, Yin Wang, Dun‐Hua Zhou, Jian‐Pei Fang, Lu‐Hong Xu

**Affiliations:** ^1^ Department of Pediatrics Sun Yat‐Sen Memorial Hospital, Sun Yat‐Sen University Guangzhou Guangdong Province China; ^2^ Guangdong Provincial Key Laboratory of Malignant Tumor Epigenetics and Gene Regulation Sun Yat‐Sen Memorial Hospital, Sun Yat‐Sen University Guangzhou Guangdong Province China; ^3^ Department of Clinical Laboratory Sun Yat‐Sen Memorial Hospital, Sun Yat‐Sen University Guangzhou Guangdong Province China

**Keywords:** 11q23/*KMT2A*, clinical outcome, gene mutations, pediatric acute myeloid leukemia, sequencing

## Abstract

**Background:**

Alterations of 11q23/KMT2A are the most prevalent cytogenetic abnormalities in acute myeloid leukemia (AML) and the prognostic significance of 11q23/KMT2A‐rearranged AML based on various translocation partners varies among different studies. However, few studies evaluated the molecular characteristics of 11q23/KMT2A‐rearranged pediatric AML. We aim to analyze the mutational landscape of 11q23/KMT2A‐rearranged AML and assess their prognostic value in outcomes.

**Methods:**

The mutational landscape and clinical prognosis of 105 children with 11q23/KMT2A‐rearranged AML in comparison with 277 children with non‐11q23/KMT2A‐rearranged AML were analyzed using publicly accessible next‐generation sequencing data from Therapeutically Applicable Research to Generate Effective Treatments (TARGET) dataset.

**Results:**

Pediatric AML patients with 11q23/KMT2A‐rearrangements harbored a low number of mutations (Median, 1 mutation/patient, range, 1‐22), 58% of which involved in RAS pathway mutations (KRAS, NRAS, and PTPN11) and 10.5% of which comprised of SETD2 mutations. Compared with non‐11q23/KMT2A‐rearranged AML, the incidence of KRAS (32.4% vs. 10.1%, P〈0.001) and SETD2 (10.5% vs. 1.4%, P=0.001) gene mutations in 11q23/KMT2A‐rearranged AML was significantly higher. Both KRAS and SETD2 mutations occurred more often in t(10;11)(p12;q23). KRAS mutations were correlated with worse 5‐year event‐free survival [EFS] (Plog‐rank = 0.001) and 5‐year overall survival [OS] (Plog‐rank = 0.009) and the presence of SETD2 mutations increases the 5‐year relapse rate (PGray = 0.004). Multivariate analyses confirmed KRAS mutations in 11q23/KMT2A‐rearranged AML as an independent predictor for poor EFS (hazard ratio [HR] = 2.10, P=0.05) and OS (HR = 2.39, P=0.054).

**Conclusion:**

Our findings show that pediatric patients with 11q23/KMT2A rearrangements have characteristic mutation patterns and varying clinical outcomes depending on different translocation partners, which could be utilized to develop more accurate risk stratification and tailored therapies.

## Graphical abstract

For 11q23/*KMT2A*‐rearranged AML pediatric patients with FAB‐M5 morphology, patients with *t*(9;11)(p22;q23) demonstrated improved outcomes compared to those with other 11q23/*KMT2A* rearrangements. Patients with 11q23/*KMT2A* rearrangements have characteristic mutation patterns and varying clinical outcomes depending on different translocation partners.

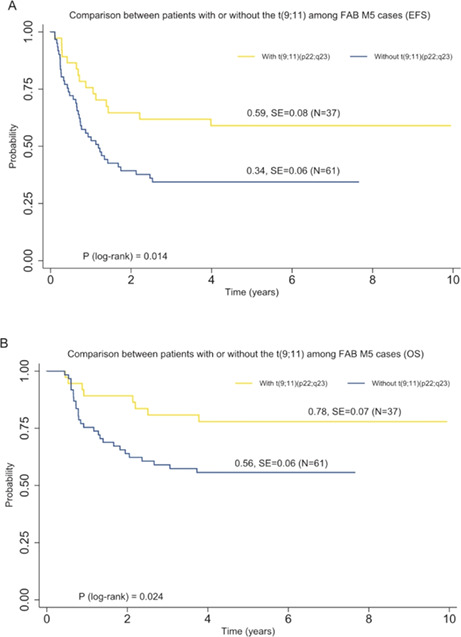

## 1 INTRODUCTION

Acute myeloid leukemia (AML) is a heterogeneous malignancy frequently present with recurrent cytogenetic and genetic abnormalities.^1^, ^2^ AML with alterations of *KMT2A* gene (Lysine [K]‐specific methyltransferase 2A, also known as *MLL* gene), located at 11q23, accounts for 15% to 20% of all childhood leukemias and are of great interest because of their distinct clinical and biological features.^3^ Morphologically, 11q23*/KMT2A*‐ rearranged AML is frequently classified as French‐American‐British (FAB) M4 and M5. To date, over 90 different 11q23/*KMT2A* rearrangements have been identified. The majority of 11q23/*KMT2A* rearrangements were seen in one of six common translocation partners: *t*(4;11)(q21;q23), *t*(6;11)(q27;q23), *t*(9;11)(p22;q23), *t*(10;11)(p12;q23), *t*(11;19)(q23;p13.1), *t*(11;19)(q23;p13.3).^4^, ^5^ Depending on the specific 11q23/*KMT2A* alterations, 11q23/*KMT2A* rearrangements correlate to a certain gene expression profile and prognoses. Fusion proteins of 11q23/*KMT2A*‐rearrangements dysregulate the expression of *HOX* cluster genes, contributing to self‐renewal potential in leukemic cells.^6^, ^7^, ^8^

*Over the past decades*, *only modest improvement in t*reatment *outcomes* for pediatric AML had been made in part because of a scarcity of targeted therapies and the slow development of immunotherapy.^9^ Even though recent clinical trials have identified novel targets such as menin inhibitor in 11q23*/KMT2A*‐ rearranged AML to impair leukemia growth and proliferation, comprehensive genome analyses provide a more thorough biological understanding for 11q23/*KMT2A*‐rearranged AML, which contributes to the exploration of targeted therapy.^10^, ^11^ Recent studies by Lavallée et al. and Bill et al. identified a high frequency of RAS pathway mutations in adult AML with 11q23/*KMT2A*‐rearrangements.^12^, ^13^ However, due to the relatively low mutation rate in 11q23/*KMT2A*‐rearranged AML, mutational characteristics of pediatric AML patients with 11q23/*KMT2A* rearrangements are rarely studied and most collaborative *cohort groups are too small for meaningful comprehensive molecular analyses of these patients. In addition*, *the prognostic value of gene mutations in* 11q23/*KMT2A*‐rearranged AML *is currently poorly understood*.

Moreover, clinical evidence indicates that the translocation partners of 11q23/*KMT2A* are a primary determinant of outcome. Previous studies demonstrated *t*(9;11)(p22;q23) was correlated with an excellent prognosis albeit several studies could not validate these results.^14^, ^15^, ^16^, ^17^, ^18^, ^19^ *Thus*, *there is a pressing need to identify the mutational landscape of* 11q23/*KMT2A*‐rearranged *pediatric AML and evaluate the* prognostic effect *of gene mutations and various* 11q23/*KMT2A translocation partners on a large scale*.

To address this demand, we analyzed the baseline characteristics and mutational data of 105 pediatric AML patients with 11q23/*KMT2A* rearrangements. Further evaluation of gene mutations combined with clinical outcomes based on different 11q23/*KMT2A* translocation partners may also contribute to the refinement of risk stratification by genetics.

## 2 PATIENTS AND METHODS

### 2.1 Study population

Data on pediatric AML patients were collected from the Therapeutically Applicable Research to Generate Effective Treatments (TARGET) program on 4 May 2021 (available at https://ocg.cancer.gov/programs/target/data‐matrix). In total, 493 pediatric patients under the age of 18 years with de novo AML between 2004 and 2010 were included in our study. Clinical features at the time of diagnosis (e.g., age, gender, white blood cell [WBC] count, cytogenetic abnormalities, and FAB classification) were obtained in clinical data file at TARGET data matrix. All pediatric patients were treated on the Children's Oncology Group (COG) protocol AAML03P1 and AAML0531.^20^, ^21^ Hematopoietic Stem Cell Transplantation (HSCT) was considered in the first complete remission for pediatric AML patients with matched‐related donors.

### 2.2 Data on cytogenetics and molecular characteristics

Cytogenetic analysis of pretreatment bone marrow samples was performed by G‐banded karyotyping; Fluorescence in situ hybridization (FISH) and reverse‐transcribed polymerase chain reaction (RT‐PCR). The International System for Human Cytogenetic Nomenclature (ISCN) was used to designate karyotypes. The mutational status of pediatric AML patients was determined centrally at the Complete Genomics sequencing platform employing high‐density DNA nanoarrays.

### 2.3 Statistical analyses

Chi‐square test was conducted to compare the frequencies of categorical variables and Mann–Whitney U test was used to compare continuous variables. Complete remission (CR) was defined as having fewer than 5% marrow blasts. Event‐free survival (EFS) was calculated from the initiation of primary treatment until the occurrence of the first event or loss to follow‐up. Overall survival (OS) was measured after the start of initial therapy to death from any cause or the point of last follow‐up. The cumulative incidence of *relapse* (*CIR*) was defined as the time from the achievement of CR until the occurrence of first *relapse*. The Kaplan–Meier model was applied to calculate 5‐year EFS and 5‐year OS, which were compared using log‐rank test. 5‐year CIR was estimated by the Fine‐Gray subdistribution hazard model and differences were analyzed by Gray's test. For multivariable analysis, the Cox proportional hazard regression model was utilized. The Cox regression model included factors that were clinically significant and statistically significant at the level of 0.05 in univariate analysis. *p* values of 0.05 or less were considered statistically significant. All analyses were carried out using R software version 4.0.5 (R Foundation for Statistical Computing).

## 3 RESULTS

### 3.1 Clinical characteristics

**FIGURE 1:**
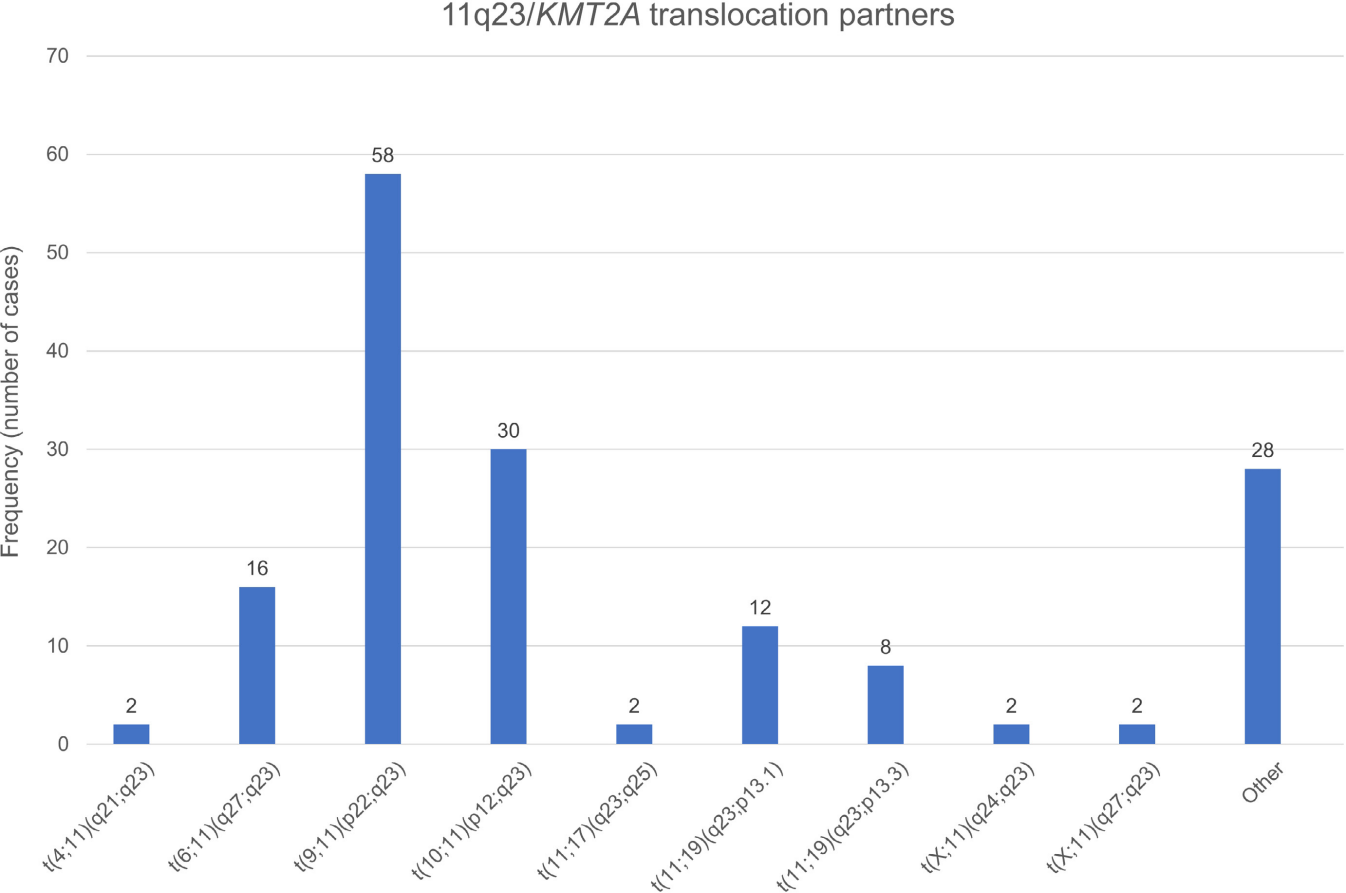
Frequency (number of cases) of pediatric patients with 11q23/*KMT2A*‐rearranged AML categorized by translocation partners.

Our study cohort consists of 493 pediatric de novo AML patients. The clinical characteristics of 160 patients with 11q23/*KMT2A* rearrangements and 333 patients without 11q23/*KMT2A* rearrangements are shown in Table 1. There were no differences in gender distribution, WBC at diagnosis, treatment protocol, and complete remission achievement between patients with 11q23/*KMT2A* rearrangements and without 11q23/*KMT2A* rearrangements. Nevertheless, 11q23/*KMT2A*‐rearranged AML patients at diagnosis had significantly lower median age compared with non‐11q23/*KMT2A*‐rearranged AML patients (Median, 3.1 vs.10.4 years, *p* < 0.001). The prevalence of pediatric AML patients with 11q23/*KMT2A* rearrangements was highest among children under the age of 2 years. The median blast percentage in pretreatment bone marrow samples was significantly higher in 11q23/*KMT2A*‐rearranged AML patients (79.5% vs. 66.0%, 11q23/*KMT2A*‐rearranged AML vs. non‐11q23/*KMT2A*‐rearranged AML, *p* < 0.001). Patients with 11q23/*KMT2A*‐rearrangement was strongly correlated with FAB classification subtype M5 (71.5%, *p* < 0.001). Of the 160 cases, the most frequent translocation partners of 11q23/*KMT2A* abnormality were *t*(9;11)(p22;q23) (*n* = 58; 36.3%), *t*(10;11)(p12;q23) (*n* = 30; 18.8%); *t*(6;11)(q27;q23) (*n* = 16; 10.0%)；*t*(11;19)(q23;p13.1) (*n* = 12; 7.5%); *t*(11;19)(q23;p13.3) (*n* = 8; 5.0%) (Figure 1). The presence of additional cytogenetic abnormalities (ACAs) is defined as additional chromosomal aberrations detected by conventional cytogenetic analysis. Even though no significant differences of additional abnormalities distribution were observed among different translocation partners, ACAs in *t*(9;11)(p22;q23) were higher than other groups and contain patients with Trisomy 21. The median age of children with the presence of ACAS is 3.4 years old (data not shown).

**TABLE 1:** Clinical characteristics of pediatric patients with or without 11q23/KMT2A‐rearranged AML

	Total	With 11q23/KMT2A‐rearranged	Without 11q23/KMT2A‐rearranged	*p*‐value
*N*	493	160	333	
Gender, *n* (% males)	255 (51.7)	77 (48.1)	178 (53.5)	0.268
Age, median (year)	8.7	3.1	10.4	<0.001
Less than 2 years, *n* (%)	103 (20.9)	67 (41.9)	37 (10.8)	
2 to 9 years, *n* (%)	171 (34.7)	51 (31.9)	119 (36.0)	
10 or more years, *n* (%)	219 (44.4)	42 (26.3)	177 (53.2)	
WBC, ×109/L, Median (range)	40.5 (0.8–610.0)	42.9 (0.8–610.0)	39.6 (1.0–447.3)	0.647
Median blast in BM, % (range)	71.0 (3–100)	79.5 (6–99)	66.0 (3–100)	<0.001
Central nervous system (CNS) involvement	34 (6.9)	7 (4.4)	27 (8.1)	0.126
FAB classification, *n* (%)				<0.001
M0	7 (1.6)	2 (1.5)	5 (1.7)	
M1	33 (7.7)	5 (3.6)	28 (9.6)	
M2	125 (29.2)	5 (3.6)	120 (41.2)	
M4	134 (31.3)	23 (16.8)	111 (38.1)	
M5	109 (25.5)	98 (71.5)	11 (3.8)	
M6	4 (0.9)	—	4 (1.4)	
M7	16 (3.7)	4 (2.9)	12 (4.1)	
FLT3‐ITD, *n* (%)				<0.001
Internal tandem duplication	51 (10.3)	4 (2.5)	47 (14.1)	
Wild type	440 (89.2)	155 (96.9)	285 (85.6)	
FLT3‐TKD, *n* (%)				0.357
Mutated	27 (5.5)	11 (6.9)	16 (4.8)	
Wild type	463 (93.9)	149 (93.1)	314 (94.3)	
WT1, *n* (%)				0.004
Mutated	27 (5.5)	2 (1.3)	25 (7.5)	
Wild type	461 (93.5)	157 (98.1)	304 (91.3)	
Treatment protocol, *n* (%)				0.239
AAML03P1	55 (11.2)	14 (8.8)	41 (12.3)	
AAML0531	438 (88.8)	146 (91.3)	292 (87.7)	
CR status at end of course 1				0.153
CR, *n* (%)	379 (76.9)	118 (74.2)	261 (78.4)	
Not CR, *n* (%)	102 (20.7)	35 (22.0)	67 (20.1)	
Death, *n* (%)	9 (1.8)	6 (3.8)	3 (0.90)	
CR status at end of course 2				0.465
CR, *n* (%)	433 (87.8)	138 (87.9)	295 (88.6)	
Not CR, *n* (%)	39 (7.9)	13 (8.3)	26 (7.8)	
Death, *n* (%)	11 (2.2)	6 (3.8)	5 (1.5)	
HSCT in 1st CR, *n* (%)				0.101
Yes	45 (9.1)	21 (13.1)	24 (7.2)	
No	411 (83.4)	128 (80)	283 (85.0)	

### 3.2 Mutational landscape of pediatric 11q23/ *KMT2A* ‐rearranged AML

**FIGURE 2:**
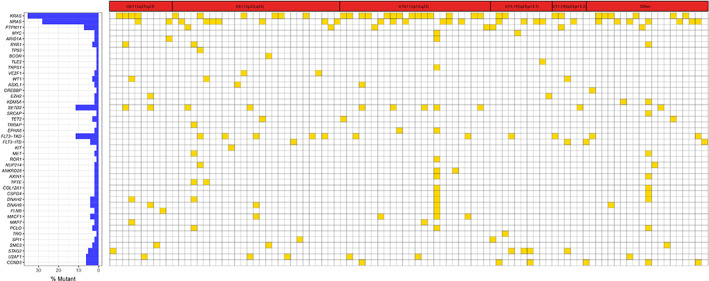
Mutational landscape of 11q23/*KMT2A*‐rearranged pediatric AML patients based on recurrent translocation partners. Each column represents a single patient, and each row represents a particular gene. Yellow, mutated; white, wild‐type. The frequencies of these gene mutations are provided on the left column.

105 children with 11q23/*KMT2A*‐rearranged AML and 277 children with non‐11q23/*KMT2A*‐rearranged AML were available for comprehensive molecular analysis of 177 recurrently mutated genes by targeted capture sequencing. In 11q23/*KMT2A*‐rearranged AML, patients harbored at least one mutation (Median, 1 mutation/patient, range, 1–22) and mutations were found in 81 genes (Figure 2). The most common mutated genes were *KRAS* (*n* = 34, 32.4%), *NRAS* (*n* = 27, 25.7%), *SETD2* (*n* = 11, 10.5%), *FLT3‐TKD* (*n* = 11, 10.5%), and *PTPN11* (*n* = 7, 6.7%). Among non‐11q23/*KMT2A*‐rearranged AML, the recurrent gene mutations were *NRAS* (*n* = 105, 37.9%), *KIT* (*n* = 72, 26.0%), *FLT3‐ITD* (*n* = 47, 17.0%), *WT1* (*n* = 36, 13.0%), and *KRAS* (*n* = 28, 10.1%). In summary, the incidence of *KRAS* (32.4% vs. 10.1%, *p* < 0.001) and *SETD2* (10.5% vs. 1.4%, *p* = 0.001) gene mutations was substantially higher in 11q23/*KMT2A*‐rearranged AML pediatric patients than non‐11q23/*KMT2A*‐rearranged AML pediatric patients. However, mutations in *KIT*, *FLT3‐ITD*, and *WT1* genes were less common in 11q23/*KMT2A*‐rearranged AML compared to non‐11q23/*KMT2A*‐rearranged AML (*p* < 0.001). No significant patterns of cooperative or mutually exclusive gene mutations were observed in 11q23/*KMT2A*‐rearranged AML cohort. Thus, our data indicated that mutations in RAS pathway involving *KRAS*, *NRAS*, and *PTPN11* genes were the most common *genetic* alterations observed in 11q23/*KMT2A*‐rearranged AML pediatric patients.

**FIGURE 3:**
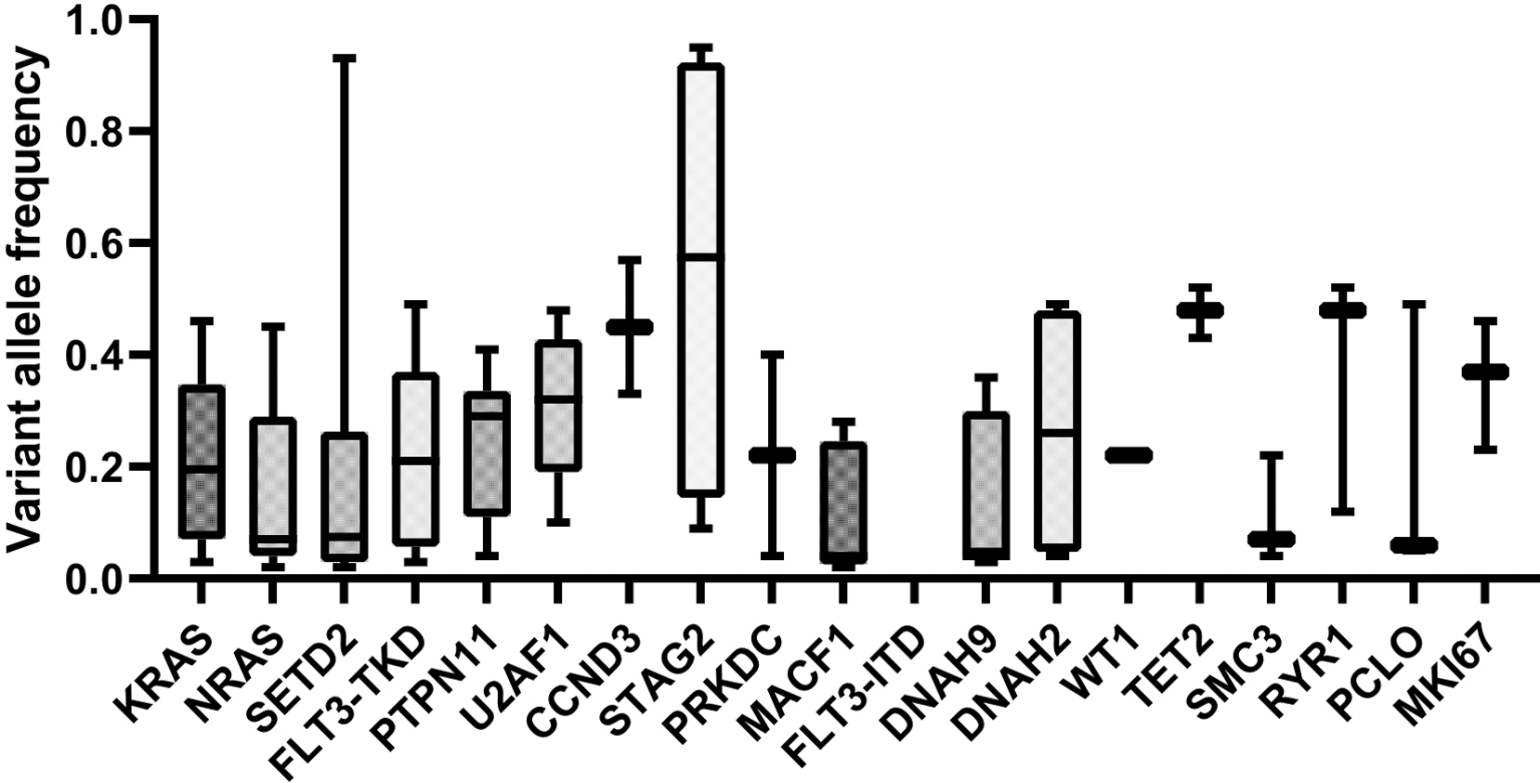
The variant allele frequencies (VAFs) of the 20 most common mutated genes in pediatric 11q23/*KMT2A*‐rearranged AML.

Further, we compared the RAS pathway mutations and *SETD2* mutations among various 11q23/*KMT2A* translocation partners and observed that *KRAS* mutations occurred with a significantly higher frequency in patients with *t*(10;11)(p12;q23) than in patients with other 11q23/*KMT2A* rearrangements (56.0% vs. 22.5%, *p* = 0.001) while *NRAS* and *PTPN11* mutations were not significantly different. *SETD2* gene was more frequently mutated in patients with *t*(10;11)(p12;q23) (5 of 25; 20.0%). To evaluate the pathogenesis of gene mutations during leukemogenesis, we investigated the variable allele frequencies (VAFs) of the 20 most prevalent gene mutations that occurred in 11q23/KMT2A‐rearranged AML (Figure 3). Among the recurrent gene mutations in children with 11q23/*KMT2A*‐rearranged AML, those with higher VAFs (>0.3) gene mutations were *U2AF1*, *CCND3*, *STAG2*, *TET2*, *RYR1*, and *MKI67*, which presumably occur earlier during leukemogenesis. On the contrary, low VAFs (<0.3) which may represent later events in a subset of leukemic cells and accelerate disease progression were found in RAS pathway genes, *SETD2*, and *FLT3‐TKD*.

### 3.3 Clinical outcome of 11q23/ *KMT2A* ‐rearranged AML

All pediatric patients had a median follow‐up time of 4.7 years. Among 493 pediatric AML patients, 55 received protocol AAML03P1 and 438 were treated with protocol AAML0531 (11.2% vs. 88.8%, *p* = 0.239). For the cohort of 11q23/*KMT2A*‐rearranged AML pediatric patients, the overall CR rate was 74.2% at the end of the first phase of chemotherapy and 87.9% at the second phase of chemotherapy with no significant differences in CR rates between pediatric AML patients with 11q23/*KMT2A*‐rearrangements and without 11q23/*KMT2A* ‐rearrangements. Similarly, there were no significant differences observed in CR rates among recurrent 11q23/*KMT2A* translocation partners at the end of the first phase of chemotherapy (*p* = 0.352) and the second phase of chemotherapy (*p* = 0.628). Nonetheless, pediatric AML patients with 11q23/*KMT2A*‐rearrangements had worse outcomes with 5‐year EFS of 49%, 5‐year CIR of 38%, and 5‐year OS of 67% compared to pediatric AML patients without 11q23/*KMT2A*‐rearrangements (Table 2).

**TABLE 2:** Survival estimates of prognostic factors in 11q23/*KMT2A*‐rearranged pediatric AML, including subgroups based on translocation partners

	% EFS (SE)	*P* (log‐rank)	% CIR (SE)	*P* (Gray)	% OS (SE)	*P* (log‐rank)
All	49 (23)		38 (2)		67 (2)	
11q23/KMT2A rearrangement						
With 11q23/KMT2A‐rearranged	40 (4)	<0.001	46 (4)	0.004	59 (4)	0.004
Without 11q23/KMT2A‐rearranged	54 (28)		34 (3)		70 (3)	
Translocation partners						
*t*(6;11)(q27;q23)	13 (8)	<0.001	69 (13)	0.015	25 (11)	0.067
*t*(9;11)(p22;q23)	57 (7)		33 (6)		71 (6)	
*t*(10;11)(p12;q23)	23 (8)		60 (9)		57 (9)	
*t*(11;19)(q23;p13.1)	64 (14)		17 (11)		64 (15)	
*t*(11;19)(q23;p13.3)	13 (12)		50 (21)		50 (18)	
Other 11q23/KMT2A rearrangements	39 (8)		53 (5)		53 (9)	
*t*(9;11)(p22;q23) subgroup						
*t*(9;11)(p22;q23)	57 (7)	<0.001	33 (6)	0.003	71 (6)	0.001
Other 11q23/KMT2A rearrangements combined	31 (5)		53 (5)		52 (5)	
Without 11q23/KMT2A‐rearranged	54 (28)		34 (3)		70 (3)	
Age						
Less than 2 years	41 (6)	0.154	42 (6)	0.662	70 (6)	0.024
2 to 9 years	49 (7)		45 (7)		58 (7)	
10 or more years	29 (7)		52 (8)		43 (8)	
Additional cytogenetic aberrations						
Absent	42 (5)	0.181	48 (4)	0.313	65 (5)	0.028
Present	37 (6)		33 (11)		51 (6)	
KRAS mutation						
11q23/KMT2A‐rearranged AML with KRAS mutant	21 (7)	0.001	56 (9)	0.080	41 (8)	0.009
11q23/KMT2A‐rearranged AML with KRAS wildtype	45 (5)		41 (6)		64 (4)	
NRAS mutation						
11q23/KMT2A‐rearranged AML with NRAS mutant	41 (10)	0.827	52 (10)	0.427	63 (9)	0.577
11q23/KMT2A‐rearranged AML with NRAS wildtype	40 (4)		44 (5)		58 (4)	
SETD2 mutation						
11q23/KMT2A‐rearranged AML with SETD2 mutant	18 (12)	0.193	82 (13)	0.004	46 (15)	0.354
11q23/KMT2A‐rearranged AML with SETD2 wildtype	42 (4)		42 (5)		60 (41)	
Allogeneic HSCT						
11q23/KMT2A‐rearranged AML with HSCT	52 (12)	0.219	45 (12)	0.284	63 (11)	0.707
11q23/KMT2A‐rearranged AML without HSCT	44 (5)		53 (5)		62 (44)	

*Note*: EFS, 5‐year event‐free survival estimates; CIR, indicates 5‐year cumulative incidence of relapse; OS, 5‐year overall survival estimate; *P* (Gray), *P* value from the Gray test; and *P* (log‐rank), *P* value from log‐rank test.

**FIGURE 4:**
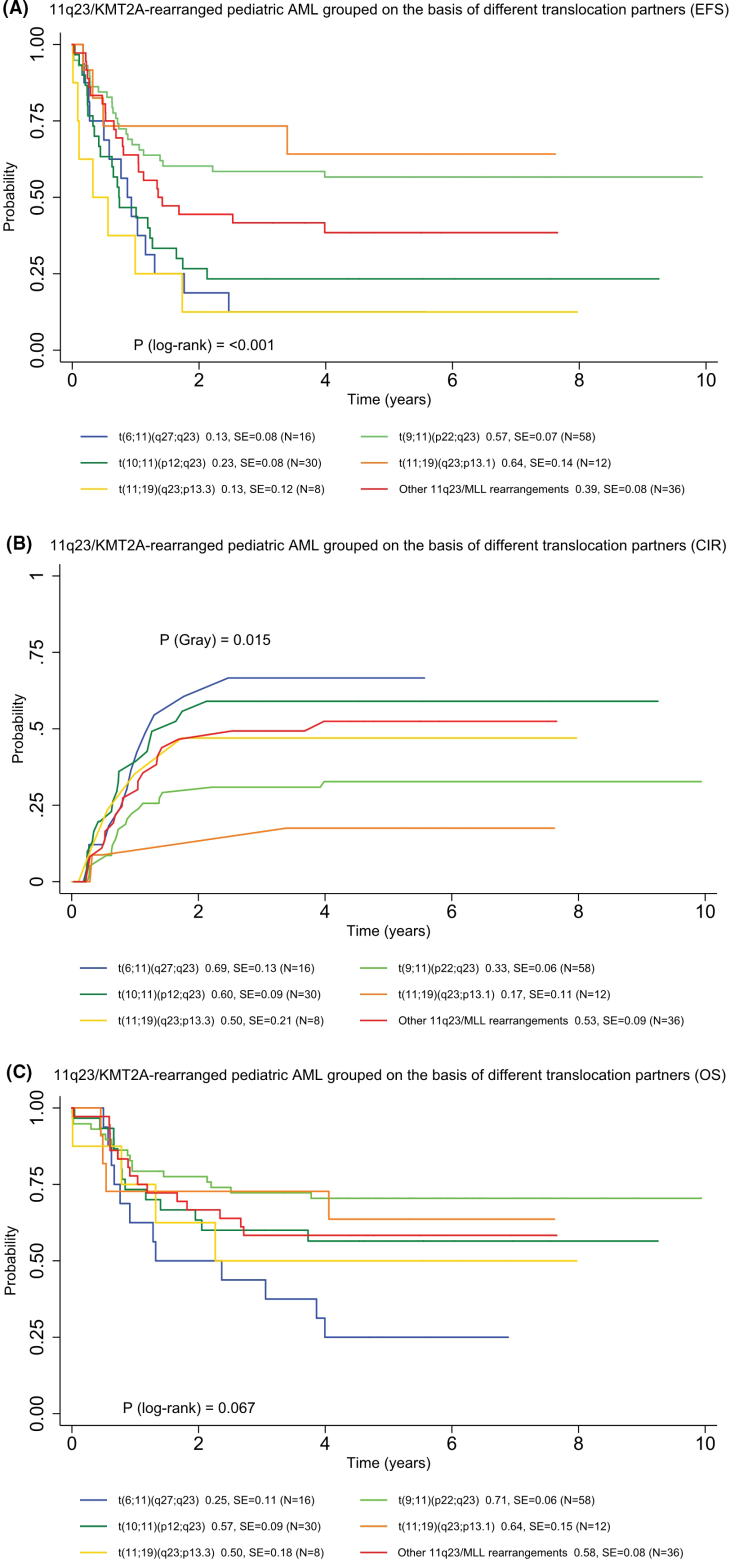
Clinical outcomes of 11q23/KMT2A‐rearranged pediatric AML grouped based on most common translocation partners. (A) 5‐year EFS. (B) 5‐year CIR. (C) 5‐year OS.

**FIGURE 5:**
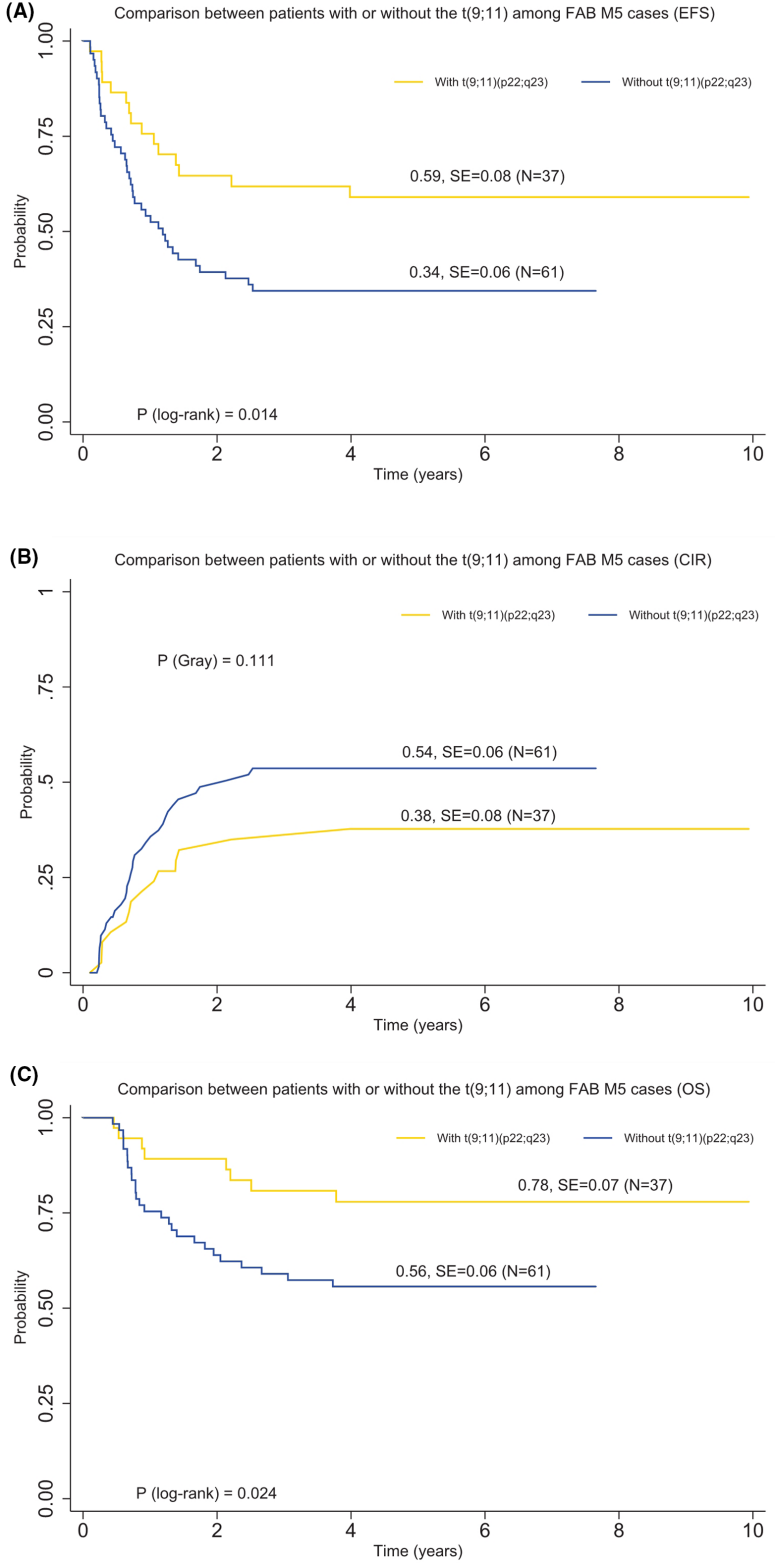
Comparison of clinical outcomes between pediatric patients with or without *t*(9;11)(p22;q23) among AML cases with FAB‐M5 morphology. (A) 5‐year EFS. (B) 5‐year CIR. (C) 5‐year OS.

The outcome among various 11q23/*KMT2A* translocation partners differed significantly (Figure 4). Pediatric patients with *t*(9;11)(p22;q23) showed better EFS (5‐year EFS, 57% vs. 31% vs.54%, *P*_log−rank_ < 0.001), CIR (5‐year CIR, 33% vs. 53% vs. 34%, *P*_Gray_ = 0.003), and OS (5‐year OS, 71% vs. 52% vs. 70%, *P*_log−rank_ = 0.001) compared with all other 11q23/*KMT2A* rearrangements combined and patients without 11q23/*KMT2A* rearrangements. Interestingly, we found that patients with *t*(11;19)(q23;p13.1) had a favorable outcome with a 5‐year EFS of 64% and a 5‐year CIR of 17%. For 11q23/*KMT2A*‐rearranged AML pediatric patients with FAB‐M5 morphology, patients with *t*(9;11)(p22;q23) demonstrated improved outcomes compared to those with other 11q23/*KMT2A* rearrangements (5‐year EFS, 59% vs. 34%, *P*_log−rank_ = 0.014; 5‐year OS, 78% vs. 56%, *P*_log−rank_ = 0.024; Figure 5).

### 3.4 Other prognostic factors

After classifying pediatric patients harboring 11q23/*KMT2A*‐rearrangements by age group, we found that patients older than 10 years old had a lower 5‐year OS when compared with patients younger than 2 years of age and patients between the ages of 2–9 years (43% vs. 70% vs. 58%, *P*_log−rank_ = 0.024; Table 2). The worse 5‐year OS of patients older than 10 years old remained significant in *t*(9;11)(p22;q23) (*P*_log−rank_ = 0.043). Moreover, we assessed the prognostic impact of addition cytogenetic aberrations on the outcome of 11q23/*KMT2A*‐rearranged AML pediatric patients. The presence of addition cytogenetic aberrations in pediatric patients tended to have a significantly shorter 5‐year OS than the other patients (51% vs. 65%, *P*_log−rank_ = 0.028). Furthermore, the prognostic impact of RAS pathway mutations was investigated. Significant worse 5‐year EFS (21% vs. 45%, *KRAS* mutated vs. *KRAS* wildtype, *P*l_og−rank_ = 0.001) and 5‐year OS (41% vs. 64%, *KRAS* mutated vs. *KRAS* wildtype, *P*_log−rank_ = 0.009) were observed for *KRAS* mutated cases with 11q23/*KMT2A*‐rearrangements. However, no impact of mutation in the *NRAS* gene on survival was observed. Of interest, the presence of *SETD2* mutations in 11q23/*KMT2A*‐rearranged AML showed a significant *increased* risk for relapse compared with *SETD2* wildtype in 11q23/*KMT2A*‐rearranged AML (82% vs. 42%, *P*_Gray_ = 0.004). Of the 160 pediatric patients with 11q23/*KMT2A*‐rearranged AML, 21 (13.1%) underwent HSCT in first remission. However, there was no significant difference seen in 5‐year EFS, 5‐year CIR, and 5‐year OS between patients who received HSCT and those who did not.

### 3.5 Multivariate analyses of pediatric patients with AML

We summarized the results of multivariate analyses for the complete cohort of patients with pediatric AML. In *multivariable* survival analysis *for EFS*, *CIR*, *and OS using* 11q23/*KMT2A‐rearrangements*, *age*, additional cytogenetic aberrations, *FLT3‐ITD*, *KRAS mutation*, *NRAS mutation*, *and HSCT as covariates*, *we revealed t*(9;11)(p22;q23) as an independent predictor with better EFS (hazard ratio [HR] = 0.56, *p* = 0.015) as well as lower CIR (HR = 0.50, *p* = 0.018). In addition, *FLT3*‐*ITD* mutation was found to be an independent predictor of poor EFS (HR = 2.01, *p* = 0.001) and higher incidence of relapse (HR = 2.23, *p* = 0.001). When the analyses were restricted to pediatric AML patients with 11q23/*KMT2A* rearrangements (Table 3), *KRAS* mutation was a significant poor prognostic factor for EFS and OS with an HR for EFS of 2.10 (*p* = 0.05) and OS of 2.39 (*p* = 0.054). We also identified pediatric patients older than 10 years old predicted poor OS (HR = 2.80, *p* = 0.001). The presence of additional cytogenetic aberrations was a significant independent predictor of OS (HR = 1.99, *p* = 0.008). Children with 11q23/*KMT2A*‐rearranged AML who underwent HSCT in the first remission tended to have longer EFS (HR = 0.58, *p* = 0.111) and OS (HR = 0.66, *p* = 0.289) than those who did not*; although the difference was not statistically significant*.

**TABLE 3:** Multivariate survival analysis of prognostic factors in 11q23/*KMT2A*‐rearranged pediatric AML

	EFS		CIR		OS	
	Hazard ratio (95% CI)	*p*	Hazard ratio (95% CI)	*p*	Hazard ratio (95% CI)	*p*
11q23/KMT2A rearrangement						
*t*(9;11)(p22;q23)	1.0 (Reference)		1.0 (Reference)		1.0 (Reference)	
Other 11q23/KMT2A rearrangements	1.56 (0.95–2.54)	0.078	1.86 (1.06–3.26)	0.030	1.63 (0.89–2.99)	0.117
Age						
Less than 2 years	1.0 (Reference)		1.0 (Reference)		1.0 (Reference)	
2 to 9 years	0.96 (0.56–1.64)	0.883	1.12 (0.62–2.01)	0.700	1.88 (0.95–3.75)	0.072
10 or more years	1.46 (0.89–2.41)	0.133	1.17 (0.67–2.03)	0.580	2.80 (1.49–5.27)	0.001
Additional cytogenetic aberrations						
Absent	1.0 (Reference)		1.0 (Reference)		1.0 (Reference)	
Present	1.52 (0.99–2.33)	0.053	1.14 (0.69–1.88)	0.610	1.99 (1.19–3.31)	0.008
FLT3‐ITD						
Wild type	1.0 (Reference)		1.0 (Reference)		1.0 (Reference)	
Internal tandem duplication	2.32 (0.67–7.96)	0.183	3.46 (1.26–9.51)	0.016	1.73 (0.38–7.90)	0.480
KRAS mutation						
Wild type	1.0 (Reference)		1.0 (Reference)		1.0 (Reference)	
Mutated	2.10 (1.26–3.50)	0.005	1.75 (0.98–3.15)	0.060	2.39 (1.29–4.46)	0.006
NRAS mutation						
Wild type	1.0 (Reference)		1.0 (Reference)		1.0 (Reference)	
Mutated	0.97 (0.54–1.75)	0.972	1.43 (0.76–2.71)	0.270	0.89 (0.42–1.85)	0.749
SETD2 mutation						
Wild type	1.0 (Reference)		1.0 (Reference)		1.0 (Reference)	
Mutated	1.32 (0.61–2.86)	0.480	1.99 (0.90–4.39)	0.088	0.75 (0.26–2.15)	0.588
Allogeneic HSCT						
No	1.0 (Reference)		1.0 (Reference)		1.0 (Reference)	
Yes	0.58 (0.29–1.14)	0.111	0.62 (0.34–1.14)	0.130	0.66 (0.30–1.43)	0.289

*Note*: CIR, indicates 5‐year cumulative incidence of relapse; EFS, 5‐year event‐free survival estimates; OS, 5‐year overall survival.

## 4 DISCUSSION

The present study analyzed a large cohort of children with de novo AML in the TARGET dataset, a collaborative initiative between the National Cancer Institute (NCI) and the Children's Oncology Group (COG). With the goal to discover novel therapeutic targets to facilitate the development of more effective treatment strategies, TARGET characterized the genomes, transcriptomes, and epigenomes of pediatric AML. Our study comprised 493 well‐annotated children from the TARGET AML dataset. Of these patients, 11.2% were enrolled onto pilot study AAML03P1 and 88.8% were randomized to the experimental arm of AAML0531. Both treatment regimens had an identical backbone, consisting of five‐course chemotherapy that includes cytarabine, daunomycin, etoposide, and Gemtuzumab Ozogamicin. AML with 11q23/*KMT2A* abnormalities is a heterogeneous subgroup of AML. It is reported that 11q23/*KMT2A* aberrations have more than 90 different translocation partners.^19^ Our data showed that the most frequent translocation in pediatric AML is *t*(9;11)(p22;q23), followed by *t*(10;11)(p12;q23), *t*(6;11)(q27;q23), *t*(11;19)(q23;p13.1), and *t*(11;19)(q23;p13.3), which is consistent with previous studies.^18^ This distribution is also analogous to adult AML.^16^

This study presents the comprehensive mutational landscape of pediatric AML patients with 11q23/*KMT2A* rearrangements using next‐generation sequencing to elucidate distinctive characteristics of pediatric 11q23/*KMT2A*‐rearranged AML with the aim of improving clinical outcomes. Gilliland et al. have postulated that both type‐I aberrations and type‐II aberrations are required for the pathogenesis of AML.^22^ Type‐I aberrations are mainly mutation hotspots involved in signaling and kinase pathway that provoke hyperproliferation of hematopoietic cells and type‐II aberrations occur as chromosomal rearrangements leading to maturation arrest. According to our data, Type‐I mutations were identified in all pediatric 11q23/*KMT2A*‐rearranged AML cases with a median of 1 mutation per patient, in line with the relatively low mutation rate in 11q23/*KMT2A*‐rearranged AML in comparison with other AML subtypes described in previous studies.^23^, ^24^ Notably, our mutational analysis revealed that 58% (61/105) of these mutations were RAS‐related mutations involved in signaling pathways (*KRAS*, *NRAS*, and *PTPN11*), similar to the high incidence of *RAS* mutations reported in adults.^12^, ^13^, ^25^ Furthermore, we unraveled the distribution of *RAS* mutations among recurrent 11q23/*KMT2A* translocation partners in pediatric AML. In adult AML, the majority of *KRAS* mutations have been reported to be present in *t*(6;11)(q27;q23).^13^, ^26^ Whereas, our data showed that *KRAS* mutations were most frequently detected in pediatric AML patients with *t*(10;11)(p12;q23) while the *mutation* frequencies of *NRAS* and *PTPN11* were similar among different translocation partners. It should be noted that few studies clearly demonstrated the prognostic value of gene mutations in 11q23/*KMT2A*‐rearranged AML. Through analyses of 160 pediatric patients with 11q23/*KMT2A*‐rearranged AML, we revealed *KRAS* mutations as an adverse prognostic predictor. The multivariate model of the complete cohort with *AML* identified that *KRAS* mutations were independent predictors of poor survival and remained independent prognostic significance in pediatric 11q23/*KMT2A*‐rearranged AML after adjustment for other risk factors. Recently, several clinical trials targeting the RAS signaling pathway with RAS inhibitors have been conducted, one of which indicated that *RAS* mutations with *t*(6;11)(q27;q23) may confer sensitivity to *mitogen‐activated protein kinase kinase (MEK) inhibition*.^27^, ^28^

In addition, we identified 10.5% of 11q23/*KMT2A*‐rearranged pediatric AML patients carried *SETD2* mutations as opposed to only 1.4% of patients without 11q23/*KMT2A*‐rearrangements and *SETD2* mutations were more prevalent in patients with *t*(10;11)(p12;q23). *SETD2*, known as an epigenetic tumor suppressor responsible for H3K36me3 modification, plays an important role in transcriptional regulation, DNA damage repair, and other cellular processes and is reported to be altered in a range of solid cancers.^29^, ^30^, ^31^ In a study by Dong et al., loss‐of‐function *SETD2* mutations in acute leukemia showed downregulation of genes involved in S and G2/M cell cycle progression, which conferred resistance to standard cytarabine‐based chemotherapy drugs.^32^ Furthermore, when checkpoint inhibitors were added to Ara‐C treatment, *SETD2* mutant AML cells resensitized to chemotherapy. Our finding of discriminative *SETD2* mutations in 11q23/*KMT2A*‐rearranged pediatric AML suggested that cytarabine‐based chemotherapy in combination with checkpoint inhibitors might provide a promising therapeutic strategy for refractory 11q23/*KMT2A*‐rearranged pediatric AML patients with chemoresistance. Few studies have explored the prognostic value of *SETD2* mutations in 11q23/*KMT2A*‐rearranged AML, particularly in pediatric patients.^33^ In a recent publication from Wang et al., the genetic polymorphism of *SETD2* in adult AML patients was evaluated for survival outcome and *SETD2* rs76208147 TT genotype performed better in overall survival.^34^ In our study, *SETD2* mutations in 11q23/*KMT2A*‐rearranged AML were strongly related with a higher relapse rate, suggesting that a more aggressive approach such as allogeneic *HSCT or novel therapeutic strategies should be considered in the early stage of treatment*. Further studies should be conducted *to clarify the biological background of SETD2* mutations and address its clinical application in pediatric AML.

The clinical outcome of 11q23/*KMT2A* rearrangements in *AML* has been investigated for more than 30 years with contradicting results reported in various studies. Nevertheless, there is consensus that the prognoses of 11q23/*KMT2A*‐rearranged AML are determined by various 11q23/*KMT2A* translocation partners. A meta‐analysis of the German AML Intergroup from eight collaborative trials suggested that *t*(v;11)(v;q23) other than *t*(9;11)(p22;q23) was associated with a poor prognosis.^17^ *Based on* 2017 ELN risk stratification by genetics,^35^ *t*(9;11)(p22;q23) was assigned to the intermediate‐risk group while other 11q23/*KMT2A* rearrangements were grouped as adverse risk. Our data confirmed that *t*(9;11)(p22;q23), distinct from other 11q23/*KMT2A* translocation partners, was an independent predictor of favorable survival in pediatric AML with a decreased rate of relapse. This was also noted in previous studies in large cohorts of adult *patients* ^14^, ^17^, ^36^ as well as pediatric patients.^15^ In contrast to this finding, some studies showed that *t*(9;11)(p22;q23) did not differ substantially from other 11q23/*KMT2A* translocation partners.^18^, ^37^ These conflicting results may attribute to the heterozygosity of the population and the variety of treatment regimens for 11q23/*KMT2A*‐rearranged AML patients enrolled in different study groups. In our study, the median age of *t*(9;11)(p22;q23) was 3.4 years and treatment protocols AAML03P1 and AAML0531 were similarly split across different translocation partners. *AML* with 11q23/*KMT2A*‐rearrangement is primarily associated with M5 *AML*. Consistent with previous studies,^15^, ^37^ FAB‐M5 pediatric patients harboring *t*(9;11)(p22;q23) demonstrated a better outcome than those without the translocation. Kuipers et al. conducted extensive gene expression analysis in 11q23/*KMT2A*‐rearranged pediatric patients and elevated IGSF4 expression was detected in *t*(9;11)(p22;q23) patients with FAB‐M5.^38^ IGSF4 overexpression in *t*(9;11)(p22;q23) patients with FAB‐M5, a cell adhesion molecule regulated by promoter methylation status, was correlated with a favorable outcome.

The present study had several limitations. Firstly, *SETD2* mutations were highlighted in our study. According to prior studies, the loss of one allele of *SETD2* does not significantly reduce H3K36me3 levels.^39^, ^40^ Hence, the clinical outcomes between monoallelic *SETD2* mutations and biallelic *SETD2* mutations may differ. However, the TARGET database does not provide detailed alterations of *SETD2* mutations to specify the loss‐of‐function feature of *SETD2* mutations. Secondly, owing to the low frequency of mutations in 11q23/*KMT2A*‐rearranged AML, the relatively small numbers of pediatric patients harboring molecular abnormalities we analyzed make validation of our results in a larger series necessary.

In conclusion, our comparisons of the mutational landscape between 11q23/*KMT2A*‐rearranged AML and non‐11q23/*KMT2A*‐rearranged *AML* revealed the relatively high frequency of mutations in the *RAS* signaling pathway and SETD2 in 11q23/*KMT2A*‐rearranged pediatric *AML*, suggesting that both gene mutations mentioned above should be incorporated into screening tests for prognostication. We also confirmed the independent prognostic influence of *t*(9;11)(p22;q23) on the clinical outcome and its correlation with FAB‐*M5 morphology*. Innovative treatment approaches should be explored based on the characteristics of 11q23/*KMT2A*‐rearranged pediatric AML found in our study.

## AUTHOR CONTRIBUTIONS

Conceptualization, K.Y.Y., J.P.F., and L.H.X.; methodology, K.Y.Y. and Y.L.; data management/analysis/ visualization, K.Y.Y. and Y.L.; data analysis and visualization of cooccurrence/ mutual exclusivity, Y.Z.Z. and Y.W.; supervision of genetic analysis, D.H.Z.; supervision, J.P.F. and L.H.X.; writing—original draft preparation, K.Y.Y.; funding acquisition, J.P.F. and L.H.X. All authors have read and approved the published version of the manuscript.

## FUNDING INFORMATION

This work was supported by the Guangdong Basic and Applied Basic Research Foundation (2021A1515010240 to J‐P F) as well as Guangdong Basic and Applied Basic Research Foundation (2020A1515010312 to L‐H X).

## CONFLICT OF INTEREST

The authors have no conflict of interest to declare.

## ETHICS APPROVAL AND CONSENT TO PARTICIPATE

This article does not contain any studies with human participants or animals performed by any of the authors.

## Data Availability

The datasets analyzed during the current study are available in the https://ocg.cancer.gov/programs/target/data‐matrix.
